# Dataset on optimizing ambulance deployment and redeployment in Fez-Meknes region, Morocco

**DOI:** 10.1016/j.dib.2022.108178

**Published:** 2022-04-13

**Authors:** Youness Frichi, Fouad Jawab, Lina Aboueljinane

**Affiliations:** a Technologies and Industrial Services Laboratory, High School of Technology of Fez, Sidi Mohamed Ben Abdellah University, Morocco; b Laboratory of Applied Mathematics and Business Intelligence, ENSMR, Rabat, Morocco; c AMIPS Research Team, EMI, Mohammed V University of Rabat, Morocco

**Keywords:** Emergency medical services, Medical transport, Ambulance location, Integer programming, Simulation, Modeling, Data, Morocco

## Abstract

Emergency Medical Services (EMS) are crucial for saving patients' life, attenuating disabilities, and improving patients' satisfaction. Optimal deployment and redeployment of ambulances over a territory reduce response times for serving emergencies. Thus, rapid interventions and transport to a hospital are guaranteed. Optimizing ambulance deployment and redeployment is achieved by conceptualizing and formulating mathematical programming models and simulation models. Mathematical models maximize the proportion of the population that can be reached by ambulance in a response time less than a threshold value. In contrast, simulation models assess a given ambulance deployment and redeployment configuration. The application of mathematical and simulation models require data related to demand areas (geographic territories), demand value at each demand area, locations of potential sites for ambulance bases, X and Y geographic coordinates of demand areas and potential sites, travel times between potential sites and demand areas, etc. All these data are essential in deciding which potential sites to choose for locating ambulance bases and how many ambulances to allocate to each base per period. Beside elaborating and constructing ambulance deployment and redeployment models, researchers in Operations Research (OR) are challenged when collecting data for executing, testing, and proving the performance of their proposed models. This paper provides data about medical transport in Morocco's Fez-Meknes region, which can be accessed at https://zenodo.org/record/6416058. They were collected from the field, estimated based on the population size, and obtained by computer programs. The dataset includes 199 demand areas and their respective demand value per ambulance type and per period, the travel times between 18, 22, 40 potential sites and the 199 demand areas per period, and the travel times between the potential sites. Also, the dataset comprises the minimum number *b* of ambulances required by each demand area for *α*-reliable coverage, which was computed using a MATLAB program. The number *b* of ambulances required by each demand area is mandatory to apply reliability models such as the MALP and the Q-MALP models. These data would be used by the research community interested in EMS, especially pre-hospital emergency issues addressed by deploying mathematical programming and simulation tools.

## Specifications Table

**Table:** 

Subject	Management Science and Operations Research
Specific subject area	Industrial Engineering, emergency medicine, ambulance location.
Type of data	Table Figure
How the data were acquired	Estimated and processed based on past interventions. Retrieved using VBA codes and Bing Map API. Computed using MATLAB programs.
Data format	Raw Processed Presented as a .xls file
Description of data collection	Data related to demand values for 199 demand areas were estimated based on the number of Emergency Medical Services interventions between 2015 and 2019 and the population size. Data concerning the location of potential sites for ambulance base location were collected from the field (Fez-Meknes region) and have included 18, 22, and 40 potential sites. Data on travel times between potential sites and demand areas were computed using VBA and Bing map API. The minimum number *b* of ambulances for *α*-reliable coverage was calculated using MATLAB.
Data source location	• City/Town/Region: Fez-Meknes region • Country: Morocco
Data accessibility	The complete dataset is provided as a Microsoft Excel file (.xls), deposited at the Zenodo repository. Repository name: Zenodo Direct URL to data: https://zenodo.org/record/6416058[1] The MATLAB codes are deposited at the Zenodo repository. Repository name: Zenodo Direct URL to the MATLAB codes: https://zenodo.org/record/6413103[2]
Related research article	Y. Frichi, F. Jawab, L. Aboueljinane, S. Boutahari, Development and comparison of two new multi-period queueing reliability models using discrete-event simulation and a simulation-optimization approach, Comput. Ind. Eng. 168 (2022) 108,068. 10.1016/j.cie.2022.108068. [3]

## Value of the Data

• Data on Emergency Medical Services (EMS) are crucial for optimizing ambulance deployment and redeployment. Indeed, researchers in Operations Research (OR) conceptualize and formulate optimization models for ambulance deployment and redeployment. They need data to solve their models, compare them with other models, and defend their proposals.
• Data presented in this article would be helpful to OR researchers interested in developing simulation and mathematical programming models for optimizing ambulance deployment and redeployment. Several articles on EMS and ambulance deployment have pointed to a lack of data as the main limitation of applying optimization models [4,5]. Making these data available and accessible would help OR researchers focus on developing their models and improving their performance rather than collecting data to test them.
• When a mathematical model is formulated, researchers need to test, compare and apply them to make them valuable. Researchers can use the provided data to study the behavior of their proposed models. They can also be used for simulation purposes. Based on the obtained results, researchers could compare the performance of their proposed models with previously developed models.

## 1 Data Description

Researchers incorporate several parameters when building simulation models or developing mathematical optimization models for ambulance deployment and redeployment [6]. These include the demand area's definition, the demand's value for each demand area, the identification of potential sites for ambulance base location, the estimation of travel times between potential sites and demand areas, etc. This section presents the data related to these parameters and the notations commonly associated with them. Note that the tables shown in the text (Tables 1 to 16) are explanatory and illustrative. All indicated data are made accessible on the Zenodo repository data https://zenodo.org/record/6416058 [1], containing complete tables.

**Table 1:** Indexes of demand areas.

Prefecture/Province	Number of Demand Areas	*i* Indexes
Fez	4 municipalities	1; 2; 3; 4
	6 boroughs	5; 6; 7; 8; 9; 10
Meknes	21 municipalities	11; 12; 13; 14; 15; 16; 17; 18; 19; 20; 21; 22; 23; 24; 25; 26; 27; 28; 29; 30; 31
…	….	….
Moulay Yacoub	11 municipalities	189; 190; 191; 192; 193; 194; 195; 196; 197; 198; 199

### 1.1 Demand

EMS demand is at the heart of ambulance deployment and redeployment models. It allows making optimal decisions about locating ambulances to cover the maximum number of requests. For this purpose, it is necessary to know the exact location of the demand areas and the value of the demand at each demand area.

#### 1.1.1 Definition of demand areas and their geographic location

**Fig. 1:**
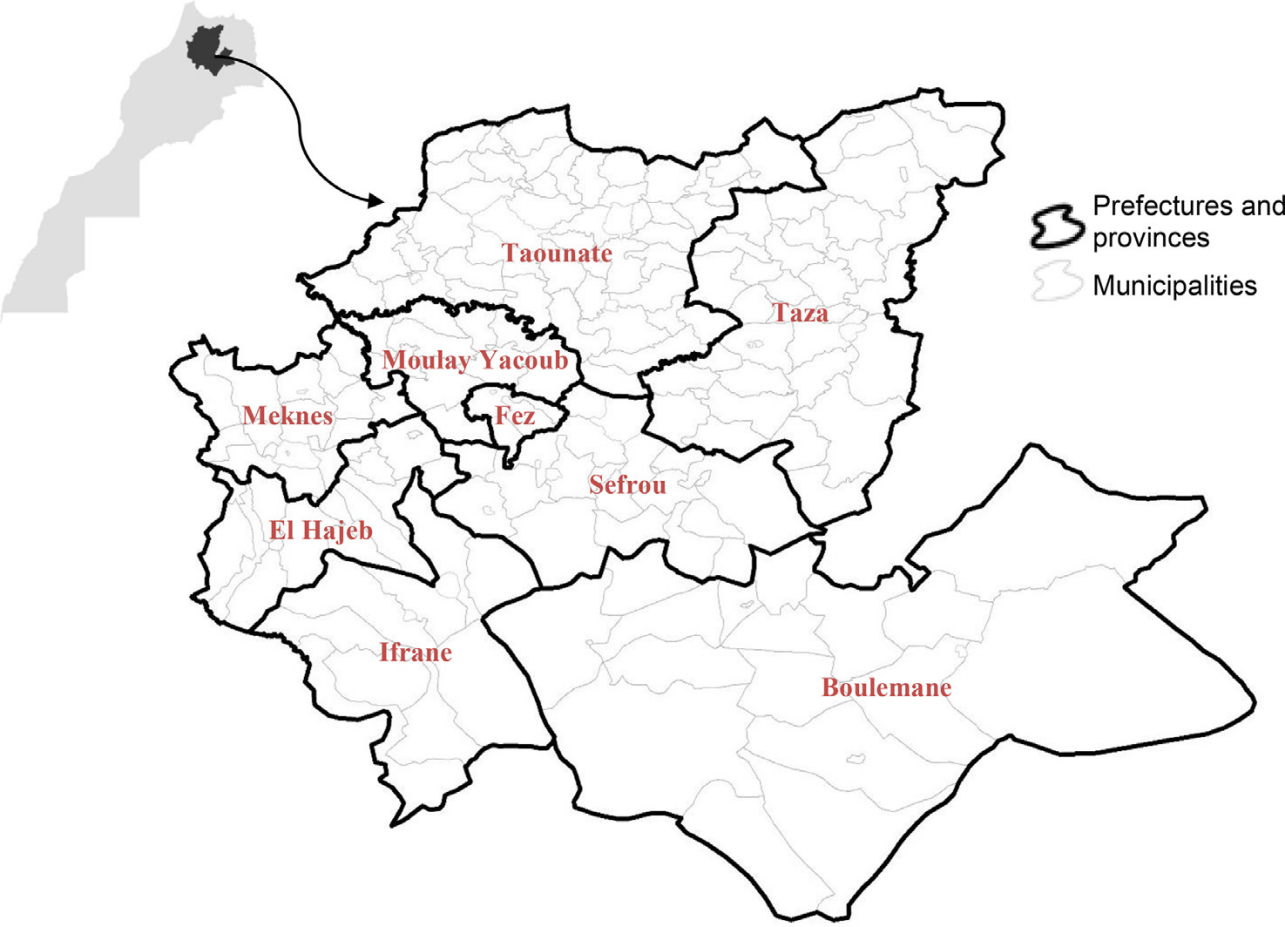
The territory of demand area, the Fez-Meknes region.

Fig. 1 presents the territory for which the dataset presented in this article was collected, computed, and made available. The territory is Morocco's Fez-Meknes region, composed of 9 prefectures and provinces. Each of them is subdivided into municipalities and boroughs, corresponding to demand areas. In total, the territory includes 199 demand areas.

#### 1.1.2 Indexes of demand areas

For modeling purposes, an index *i* is assigned to each demand area as described in Table 1. Mathematical programming models identify demand areas by their corresponding indexes and use them in equations and mathematical formulas.

#### 1.1.3 X and Y geographic coordinates of demand areas

The associated X and Y geographic coordinates for each demand area were identified and reported in Table 2. The geographic coordinates are essential because they are needed to identify demand areas and estimate travel times (see Section 2.4).

**Table 2:** X and Y geographic coordinates of demand areas.

Demand Areas Index	Latitude (X)	Longitude (Y)	Coordinates
*i* = 1	34.036100	−4.993360	34.036100,−4.993360
*i* = 2	34.013960	−4.981930	34.01396,−4.981930
…	…	…	…
*i* = 199	34.304180	−5.141180	34.304180,−5.141180

#### 1.1.4 Demand values

The value of transport demand is the number of calls received and handled by the Civil Protection Alert Processing Center in the Fez-Meknes region. In our investigation, we found only the total number of interventions in the whole territory of the prefectures and provinces. The number of interventions in each demand area is not available. In the absence of data on demand history in each demand area, researchers propose substituting demand by the size of the resident population using a scale [4].

Table 3 provides the population size in each demand area extracted from the High Commission for Planning (HCP)^1^ database, the primary data provider in Morocco.

**Table 3:** Population size in each demand area.

Demand Area	Population Size
*i* = 1	141,168
*i* *=* 2	20,311
…	…
*i* = 199	12,771

In this data paper, we suggest substituting the history of interventions with the population size in each demand area using ratios calculated by dividing the actual number of Civil Protection interventions by the population size (see Section 2.1).

Multi-period redeployment models need to dispose of the demand value at each demand area per ambulance type and time period. Most EMS systems operate with two distinct ambulance types: ALS and BLS ambulances. Table 4 provides the demand value at each demand area per ambulance type (ALS and BLS) and per period (*t* = 1 and *t* = 2). The two periods were defined based on the analysis conducted in Section 2.3.

**Table 4:** Values of demand in each demand area.

	ALS Ambulances		BLS Ambulances	
Demand Areas Index	*t* = 1	*t* = 2	*t* = 1	*t* = 2
*i* = 1	362	155	673	288
*i* = 2	52	22	97	41
…	…	…	…	…
*i* = 199	15	7	29	12

Data in Table 4 were estimated based on the population size in each demand area provided in Table 3 and using ratios described in Section 2.1. Concerning the breakdown of demands per ambulance type, we hypothesized that 65% of demands requested BLS ambulances, and 35% requested ALS ambulances. Also, 70% of demands arrive during the first period (*t* = 1) and 30% during the second period (*t* = 2).

For example, for the first line of Table 4 corresponding to the first demand area (*i* = 1), the demand value is obtained by multiplying the population size (141,168) by the ratio of 1.0470% (see Section 2.1), which gives a result of 1478 (the annual demand of demand area 1). 35% of this demand is ALS demand (517), and 75% is BLS demand (961). Then, the ALS demand during the first period (*t* = 1) is 362 (0.7 × 517) and during the second period (*t* = 2) is 155 (0.3 × 517). The same for BLS demand during the first period 673 (0.7 × 961) and during the second period 288 (0.3 × 961).

#### 1.1.5 Arrival rates

The arrival rate is the average number of requests received per hour. For ambulance deployment and redeployment optimization, the arrival rates are needed per demand area (*i* = 1, …199), per period (*t* = 1, 2), and ambulance type (ALS, BLS). They were estimated from Table 4, reporting the demand value in each demand area and using Eqs. (1)–(4).

(1) $$\lambda \;\left(i,\;t=1,\;ALS\right)=\frac{Demand\left(i,\;t=1,\;ALS\right)}{365\;\times \;15}$$

(2) $$\lambda \;\left(i,\;t=2,\;ALS\right)=\frac{Demand\left(i,\;t=2,\;ALS\right)}{365\;\times \;9}$$

(3) $$\lambda \;\left(i,\;t=1,\;BLS\right)=\frac{Demand\left(i,\;t=1,\;BLS\right)}{365\;\times \;15}$$

(4) $$\lambda \;\left(i,\;t=2,\;BLS\right)=\frac{Demand\left(i,\;t=2,\;BLS\right)}{365\;\times \;9}$$

For instance, the arrival rate λ(*i* = 1, *t* = 1, ALS) for demand area *i* = 1 is calculated by dividing the annual demand (362) from Table 4 by the duration of the period *t* = 1 in hours (15 h) multiplied by 365 days of the year.

Table 5 provides the arrival rates of demands requesting ambulances of type ALS and BLS per period.

**Table 5:** Demand arrival rates.

	ALS Ambulances		BLS Ambulances	
Demand Area Index	λ (i, *t* = 1) per hour	λ (i, *t* = 2) per hour	λ (i, *t* = 1) per hour	λ (i, *t* = 2) per hour
*i* = 1	0.06614	0.04724	0.12284	0.08774
*i* = 2	0.00952	0.00680	0.01767	0.01262
…	…	…	…	…
*i* = 199	0.00282	0.00201	0.00524	0.00374

### 1.2 Service time

When an ambulance is dispatched to serve demand, it becomes busy during an amount of time, called service time. It corresponds to the time it takes for the ambulance to get to the demand area, provide on-site care, transport the patient to a hospital, and return to the ambulance base.

Researchers developing probabilistic mathematical programming models and simulation models need to estimate the service time at each demand area. Table 6 provides service time at each of the 199 demand areas included in the dataset.

**Table 6:** Service time per demand area.

Demand Area Index	Service Time (minutes)
*i* = 1	31.4
*i* = 2	16.8
…	….
*i* = 199	117.3

### 1.3 Potential sites

Potential sites correspond to some selected demand areas that can host ambulance bases. In this dataset, we have considered three cases of potential sites. The first case corresponds to the current ambulance base sites in the Fez-Meknes region (18 potential sites). The second case adds sites with hospitals (22 potential sites). The third case includes all urban municipalities (40 potential sites). For modeling purposes, we differentiate between potential sites and demand areas. For this, we used different indices: the *i* index for the demand areas (*i* = 1, ..., 199) and the *j* index for the potential sites. For the first case of 18 potential sites *j* = 1, ... 18; the second case, *j* = 1, ... 22; the third case *j* = 1, …40. However, note that potential sites are also demand areas. Table 7 lists the potential sites considered in the three cases with their *i* and *j* indexes.

**Table 7:** Definition of potential sites.

Prefecture/ Province	18 Potential Sites	22 Potential Sites	40 Potential Sites
Fez	Agdal borough (*j* = 1; *i* = 1) Merinide borough (*j* = 2; *i* = 6)	Agdal borough (*j* = 1; *i* = 1) Saiss borough (*j* = 2; *i* = 3) Fez-Medina borough (*j* = 3; *i* = 4) Merinide borough (*j* = 4; *i* = 6)	Agdal borough (*j* = 1; *i* = 1) Municipality Mechouar Fez Jdid (*j* = 2; *i* = 2) Saiss borough (*j* = 3; *i* = 3) Fez-Medina borough (*j* = 4; *i* = 4) Jnan Lward borough (*j* = 5; *i* = 5) Merinide borough (*j* = 6; *i* = 6) Zouagha borough (*j* = 7; *i* = 7)
Meknes	Meknes municipality (*j* = 3; *i* = 11) Moulay Driss Zerhoun (*j* = 4; *i* = 15)		Meknes municipality (*j* = 8; *i* = 11) Al Machouar – Stinia (*j* = 9; *i* = 12) Boufakrane (*j* = 10; *i* = 13) Toulal (*j* = 11; *i* = 14) Moulay Driss Zerhoun (*j* = 12; *i* = 15) Ouislane (*j* = 13; *i* = 16)
…	…	…	…
Moulay Yacoub			Moulay Yacoub municipality (*j* = 40; *i* = 189)

**Fig. 2:**
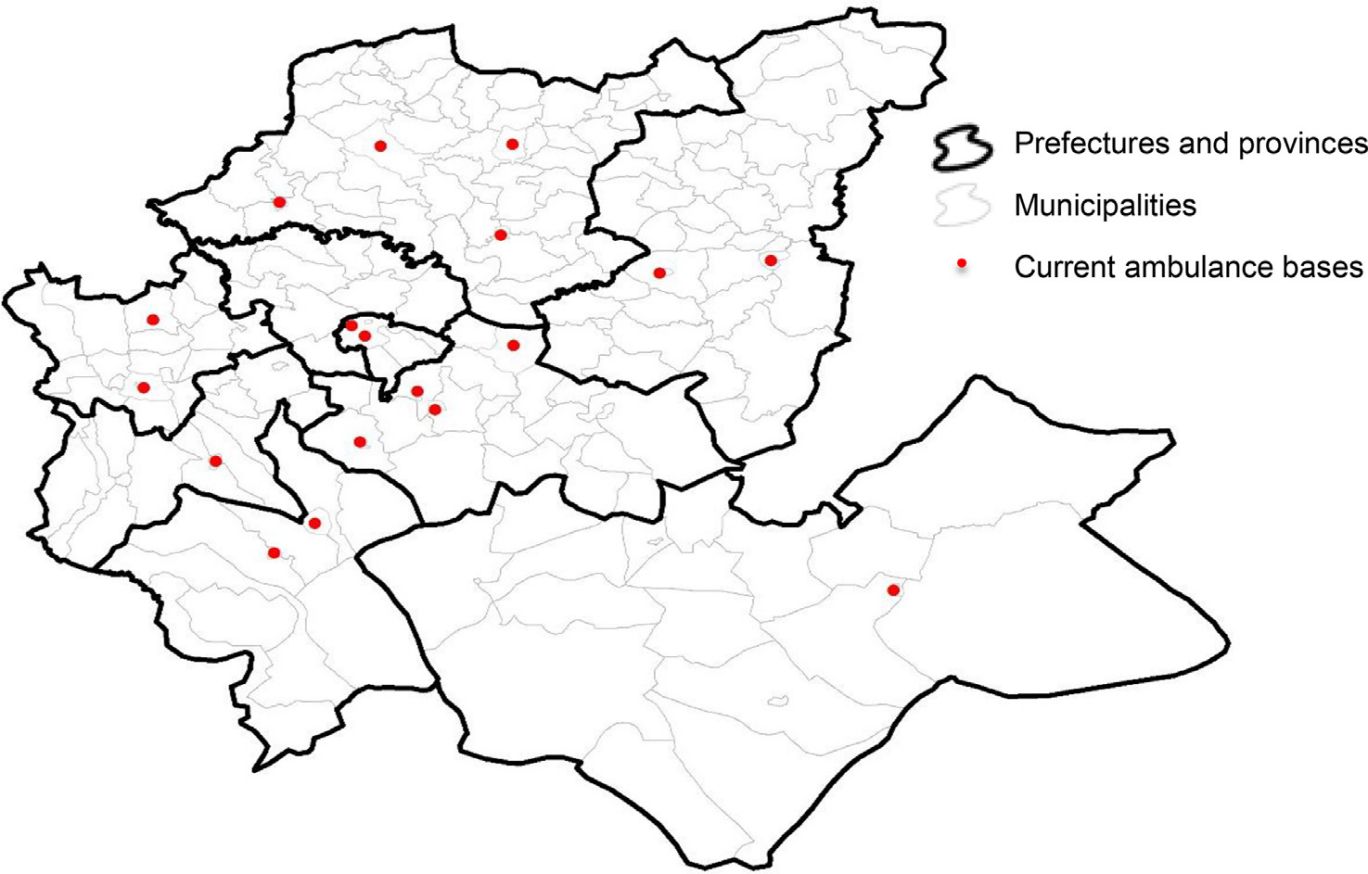
Location of the 18 potential sites.

**Fig. 3:**
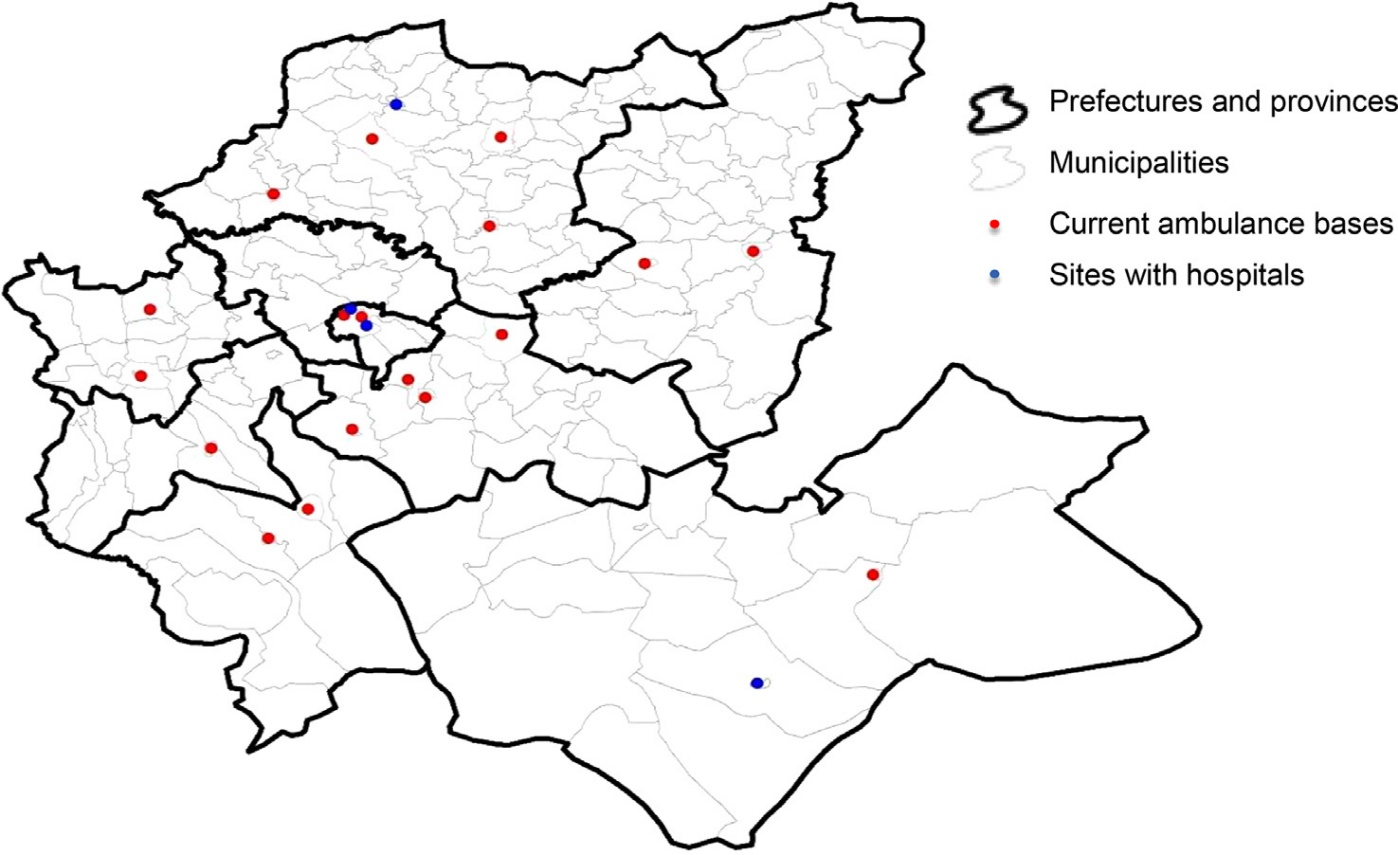
Location of 22 potential sites.

**Fig. 4:**
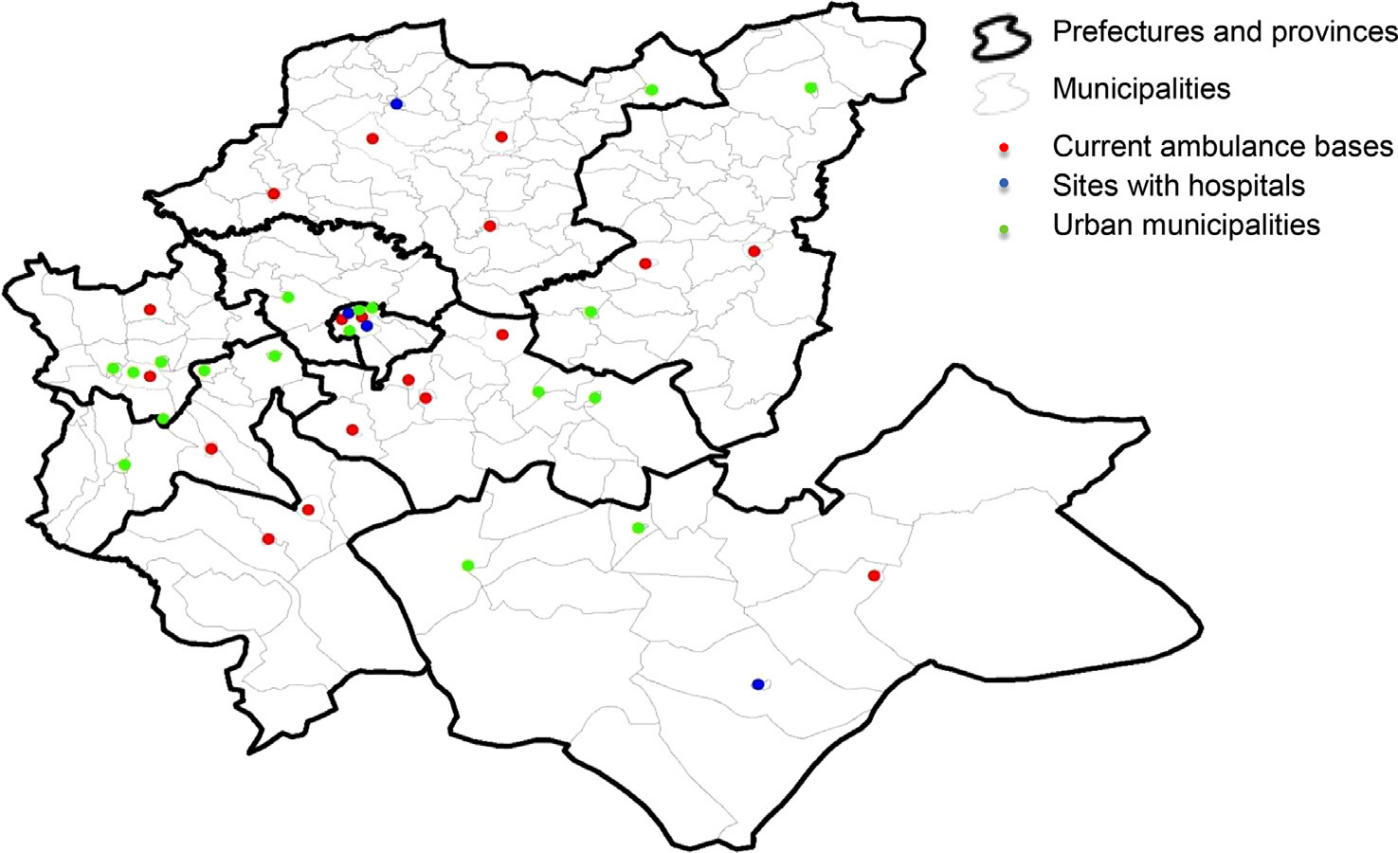
Location of 40 potential sites.

Fig. 2, Fig. 3, Fig. 4 show the locations of the 18, 22, and 40 potential sites, respectively.

### 1.4 Number of ambulances

We provide in Table 8 different options concerning the maximum number of ambulances to be deployed per type and per period. These numbers constitute the upper bound of deployed ambulances.

**Table 8:** Options of the maximum number of ambulances.

Options	Period	ALS Ambulances	BLS Ambulances	Total
Option 1	1	19	38	57
	2	19	19	38
…	…	…	…	…
Option 4	1	152	190	342
	2	114	152	266

The data in Table 8 can be used for testing purposes and analyzing the behavior of the optimization models based on the number of ambulances to be deployed.

### 1.5 Travel time

Travel times between the 18, 22, and 40 potential sites and the 199 demand areas per period are indicated in Table 9, Table 10, and Table 11, respectively.

**Table 9:** Travel times between 18 potential sites and 199 demand areas.

	Period 1			Period 2		

Demand Area Index Potential Site Index	*i* = 1	…	*i* = 199	*i* = 1	…	*i* = 199
*j* = 1	3 min	…	70 min	0	…	67 min
*j* = 2	13 min	…	67 min	10 min	…	64 min
…	…	…	…	…	…	…
*j* = 18	85 min	…	137 min	82 min	…	134 min

**Table 10:** Travel times between 22 potential sites and 199 demand areas.

	Period 1			Period 2		

Demand Area Index Potential Site Index	*i* = 1	…	*i* = 199	*i* = 1	…	*i* = 199
*j* = 1	3 min	…	70 min	0	…	67 min
*j* = 2	11 min	…	72 min	8 min	…	69 min
…	…	…	…	…	…	…
*j* = 22	85 min	…	137 min	82 min	…	134 min

**Table 11:** Travel times between 40 potential sites and 199 demand areas.

	Period 1			Period 2		

Demand Area Index Potential Site Index	*i* = 1	…	*i* = 199	*i* = 1	…	*i* = 199
*j* = 1	3 min	…	70 min	0	…	67 min
*j* = 2	10 min	…	65 min	7 min	…	62 min
…	…	…	…	…	…	…
*j* = 40	32 min	…	45 min	29 min	…	42 min

For multi-period redeployment, travel times are mandatory between each potential site and the other potential sites per period. Table 12, Table 13, Table 14 give the travel time between the 18 potential sites, 22 potential sites, and 40 potential sites.

**Table 12:** Travel times between the 18 potential sites.

	Period 1			Period 2		
Potential Sites Index	*j* = 1	…	*j* = 18	*j* = 1	…	*j* = 18
*j* = 1	3 min	…	87 min	0	…	84 min
*j* = 2	13 min	…	90 min	10 min	…	87 min
…	…	…	…	…	…	…
*j* = 18	85 min		3 min	82 min	…	0 min

**Table 13:** Travel times between the 22 potential sites.

	Period 1			Period 2		
Potential Sites Index	*j* = 1	…	*j* = 22	*j* = 1	…	*j* = 22
*j* = 1	3 min	…	87 min	0	…	84 min
*j* = 2	11 min	…	79 min	8 min	…	76 min
…	…	…	…	…	…	…
*j* = 22	85 min	…	3 min	82 min	…	0

**Table 14:** Travel times between the 40 potential sites.

	Period 1			Period 2		
Potential Sites Index	*j* = 1	…	*j* = 40	*j* = 1	…	*j* = 40
*j* = 1	3 min	…	32 min	0	…	29 min
*j* = 2	10 min	…	30 min	7 min	…	27 min
…	…	…	…	…	…	…
*j* = 40	32 min	…	3 min	29 min	…	0

### 1.6 Number of ambulances for *α*-reliable coverage

When researchers base their ambulance deployment and redeployment models on *α*-reliability models such as the MALP [7] model and the Q-MALP model [8], the number $b_{it}^{k}$ of ambulances is needed to solve these models. It refers to the minimum number of ambulances of type *k* that have to cover demand area *i* during period *t* for *α*-reliable coverage. In Table 15 we provide the $b_{it}^{k}$ corresponding to *α* equal 90%.

**Table 15:** Number $b_{\mathrm{it}}^{k}$ of ambulances for α-reliable coverage.

Index of the Demand Area	$b_{it}^{k}$ (*t* = 1, *k*=ALS)	$b_{it}^{k}$ (*t* = 2, *k*=ALS)	$b_{it}^{k}$ (*t* = 1, *k*=BLS)	$b_{it}^{k}$ (*t* = 2, *k*=BLS)
*i* = 1	8	6	14	10
*i* = 2	2	2	3	3
…	…	…	…	…
*i* = 199	2	2	2	2

#### 1.6.1 Number $b_{it}^{k\alpha_{m}}$ of ambulances for *α*_m_-reliable coverage

When models are more sophisticated and precise, the *α* reliability may vary from one demand area to another. The model developed by Frichi et al. [3] considers a variable reliability *α*_m_ ranging from *α*_1_=1% to *α*_90_=90%. For this model, the number $b_{it}^{k\alpha_{m}}$ of ambulances of type *k* required by demand area *i* during period *t* for *α*_m_ reliable coverage (*α*_1_=1%, …; *α*_90_=90%) is provided in Table 16.

**Table 16:** Number $b_{\mathrm{it}}^{k\alpha_{m}}$ of ambulances for α_m_-reliable coverage.

	$b_{it}^{k\alpha_{m}}$(*t* = 1 and *k*=ALS)				$b_{it}^{k\alpha_{m}}$(*t* = 2 and *k*=ALS)			$b_{it}^{k\alpha_{m}}$(*t* = 1 and *k*=BLS)			$b_{it}^{k\alpha_{m}}$(*t* = 2 and *k*=BLS)		
Index of the Demand Area	*α*_1_=1%	*α*_2_=2%	…	*α*_90_=90%	*α*_1_=1%	…	*α*_90_=90%	*α*_1_=1%	…	*α*_90_=90%	*α*_1_=1%	…	*α*_90_=90%
*i* = 1	1	1	…	8	1	…	6	1	…	14	1	…	10
*i* = 2	1	1	…	2	1	…	2	1	…	3	1	…	3
…	…	…	…	…	…	…	…	…	…	…	…	…	…
*i* = 199	1	1	…	2	1	…	2	1	…	2	1	…	2

## 2 Experimental Design, Materials and Methods

### 2.1 Definition of demand areas and estimation of demand values

The demand areas were extracted from the High Commission for Planning (HCP), Morocco's official producer and provider of statistics. Table 17 specifies the number of demand areas of the prefectures and provinces of the Fez-Meknes region.

**Table 17:** Number of demand areas per prefecture and province.

Prefecture/Province	Surface (Km^2^)	Number of Demand Areas
Fez	332	10 (4 municipalities and 6 boroughs)
Meknes	1 786	21
Boulemane	14 395	21
El Hajeb	2 209	16
Ifrane	3 310	10
Sefrou	4 008	23
Taounate	5 585	49
Taza	7 098	38
Moulay Yacoub	1 700	11
**Total**	**40 423**	**199**

The value of transport demand is the annual number of calls received and handled by the Civil Protection (CP) Alert Processing Center in the Fez-Meknes region. The history of transport interventions is often used to estimate the demand. This approach is widely adopted, especially since it is difficult to predict how much, where, and when transport demands will occur. Hence, we calculated a ratio corresponding to the total number of CP service interventions in each prefecture and province to its population size (Table 18).

**Table 18:** Number of interventions by province/prefecture.

Prefecture/Province	Interventions	Reference Year	Population Size	Ratio Interventions/Population
Fez	12 000	2016	1 146 088	1.0470%
Meknes	8 000	2015	827 479	0.9668%
Boulemane	2 000	2016	197 475	1.0128%
El Hajeb	1 150	2015	246 173	0.4672%
Ifrane	2 900	2015	153 771	1.8859%
Sefrou	5 000	2019	285 938	1.7486%
Taounate	1 300	2018	660 736	0.1968%
Taza	4 500	2016	526 986	0.8539%
Moulay Yacoub	850	2016	172 311	0.4933%

The ratios are then used to estimate the demand value in each demand area belonging to each prefecture and province. Indeed, the population size of each demand area was multiplied by the ratio for the prefecture/province to which it belongs.

### 2.2 Definition and location of potential sites

The first case of potential sites (18 potential sites) corresponds to the current location of the CP services in the Fez-Meknes region. The CP staff identified the 18 potential sites. The second case (22 potential sites) added to the 18 potential sites 4 other potential sites corresponding to hospitals identified by hospital managers.^2^ The third case (40 potential sites) added to the 22 potential sites 18 other potential sites corresponding to urban areas identified by the HCP.

### 2.3 Definition of time periods

**Fig. 5:**
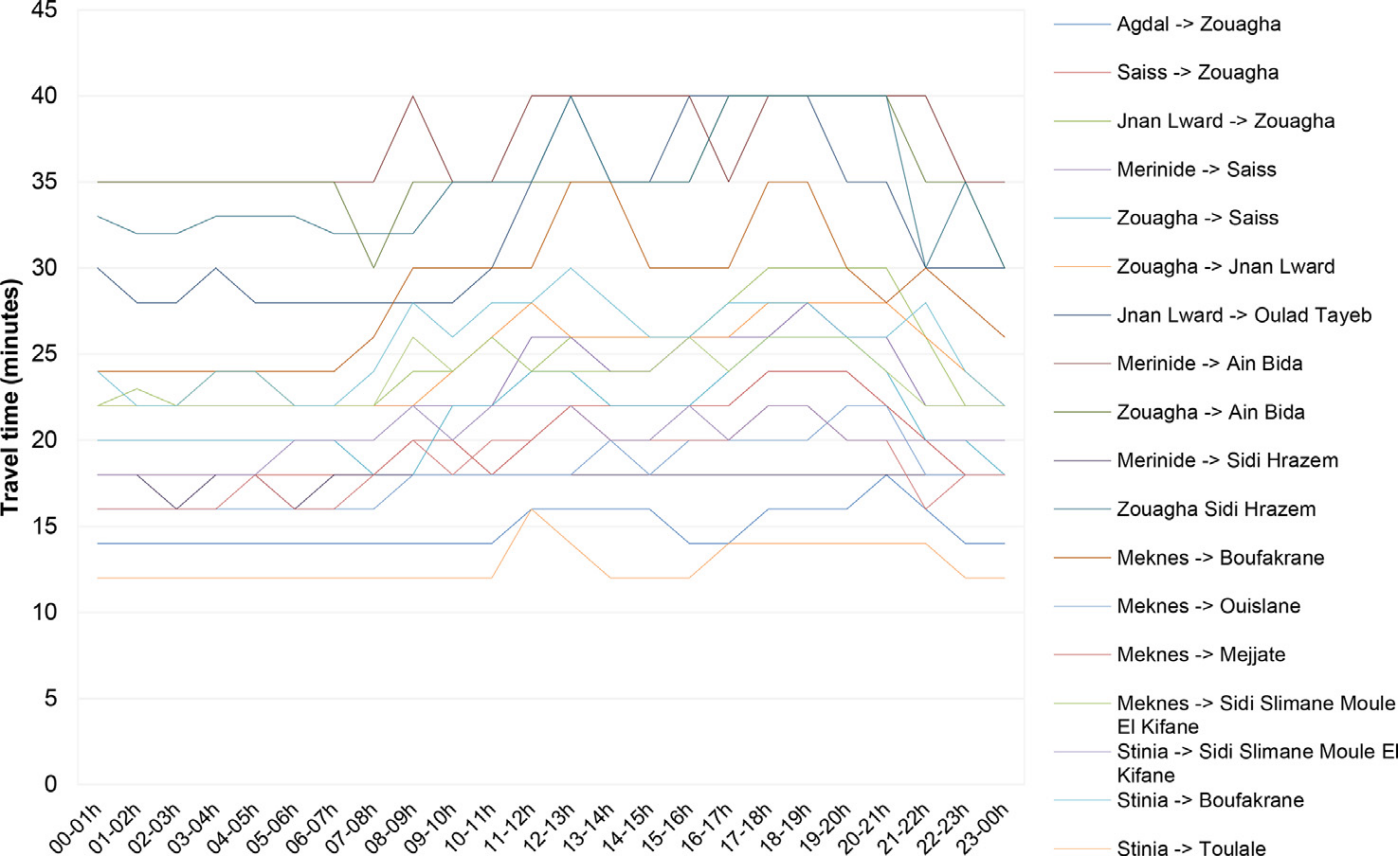
Hourly variation in travel time.

Due to the variation of travel time with traffic depending on the day time periods (i.e., peak hours), multi-period redeployment models suggest subdividing the day into homogeneous periods defined by observing the evolution of travel times. We used the Google Maps mobile application to capture the hourly variations in travel times between a sample of geographic areas in Fez and Meknes cities, the most populated urban areas in the Fez-Meknes region (Table 19). Fig. 5 shows the variation in travel time per hour of the day. It can be observed that two principal periods characterized travel times. The first period is from 7 h to 22 h and the second from 22 h to 7 h.

**Table 19:** Travel times between a set of urban areas.

Origin-destination	00–01h	01–02h	02–03h	03–04h	04–05h	05–06h	06–07h	07–08h	08–09h	09–10h	10–11h	11–12h	12–13h	13–14h	14–15h	15–16h	16–17h	17–18h	18–19h	19–20h	20–21h	21–22h	22–23h	23–00h
Agdal -> Zouagha	14	14	14	14	14	14	14	14	14	14	14	16	16	16	16	14	14	16	16	16	18	16	14	14
Saiss -> Zouagha	18	18	18	18	18	18	18	18	20	20	18	20	22	22	22	22	22	24	24	24	22	20	18	18
Jnan Lward -> Zouagha	22	23	22	24	24	22	22	22	24	24	26	24	26	26	26	26	28	30	30	30	30	26	22	22
Merinide -> Saiss	22	22	22	22	22	22	22	22	22	22	22	26	26	24	24	26	26	26	28	26	26	22	22	22
Zouagha -> Saiss	20	20	20	20	20	20	20	18	18	22	22	24	24	22	22	22	24	26	26	26	24	20	20	18
Zouagha -> Jnan Lward	22	22	22	24	24	22	22	22	22	24	26	28	26	26	26	26	26	28	28	28	28	26	24	22
Jnan Lward -> Oulad Tayeb	30	28	28	30	28	28	28	28	28	28	30	35	40	35	35	40	40	40	40	35	35	30	30	30
Merinide -> Ain Bida	35	35	35	35	35	35	35	35	40	35	35	40	40	40	40	40	35	40	40	40	40	40	35	35
Zouagha -> Ain Bida	35	35	35	35	35	35	35	30	35	35	35	35	35	35	35	35	40	40	40	40	40	35	35	30
Merinide -> Sidi Hrazem	18	18	16	18	18	16	18	18	18	18	18	18	18	18	18	18	18	18	18	18	18	18	18	18
Zouagha Sidi Hrazem	33	32	32	33	33	33	32	32	32	35	35	35	40	35	35	35	40	40	40	40	40	30	35	30
Meknes -> Boufakrane	24	24	24	24	24	24	24	26	30	30	30	30	35	35	30	30	30	35	35	30	28	30	28	26
Meknes -> Ouislane	16	16	16	16	16	16	16	16	18	18	18	18	18	20	18	20	20	20	20	22	22	18	18	18
Meknes -> Mejjate	16	16	16	16	18	16	16	18	20	18	20	20	22	20	20	20	20	22	22	20	20	16	18	18
Meknes -> Sidi Slimane Moule El Kifane	22	22	22	22	22	22	22	22	26	24	26	24	24	24	24	26	24	26	26	26	24	22	22	22
Stinia -> Sidi Slimane Moule El Kifane	18	18	18	18	18	20	20	20	22	20	22	22	22	20	20	22	20	22	22	20	20	20	20	20
Stinia -> Boufakrane	24	22	22	24	24	22	22	24	28	26	28	28	30	28	26	26	28	28	28	26	26	28	24	22
Stinia -> Toulale	12	12	12	12	12	12	12	12	12	12	12	16	14	12	12	12	14	14	14	14	14	14	12	12

### 2.4 Estimating travel time

To estimate travel times, we have used a VBA code that communicates with the Bing Map engine using an API.

### 2.5 Computation of the number $b_{it}^{k}$ of ambulances

For *α*-reliable coverage, there must be at least $b_{it}^{k}$ ambulances of type *k* covering demand area *i* during period *t*. The $b_{it}^{k}$ is expressed using queuing theory formulas of a system M/G/Z/Z. $b_{it}^{k}$ is the smallest integer satisfying Eq. (5).

(5) $$\frac{\frac{{\left(\rho_{it}^{k}\right)}^{b_{it}^{k}}}{b_{it}^{k}!}}{1+\;\rho_{it}^{k}+\frac{{\left(\rho_{it}^{k}\right)}^{2}}{2!}+\cdots +\frac{{\left(\rho_{it}^{k}\right)}^{b_{it}^{k}}}{b_{it}^{k}!}}\leq 1-\;\alpha$$

$\rho_{it}^{k}=\lambda_{it}^{k}\;/\;\mu_{it}^{k}$ is the traffic intensity, $\lambda_{it}^{k}$ is the arrival rate of requests from area *i* during period *t* requiring ambulances of type *k*, and $1\;/\;\mu_{it}^{k}$ is the average service time of ambulances of type *k* responding to requests from area *i* during period *t*. Knowing the values of $\rho_{it}^{k}$ and *α*, it is possible to determine the value of $b_{it}^{k}$. Similarly, the numbers $b_{it}^{k\alpha_{m}}$ of ambulances required to cover demand areas *i* with *α*_m_(*m* = 1, …90) reliability were computed using Eq. (6).

(6) $$\frac{\frac{{\left(\rho_{it}^{k}\right)}^{b_{it}^{k\alpha_{m}}}}{b_{it}^{k\alpha_{m}}!}}{1+\;\rho_{it}^{k}+\frac{{\left(\rho_{it}^{k}\right)}^{2}}{2!}+\cdots +\frac{{\left(\rho_{it}^{k}\right)}^{b_{it}^{k\alpha_{m}}}}{b_{it}^{k\alpha_{m}}!}}\leq 1-\;\alpha_{m}$$

To determine the values of $b_{it}^{k}$ and $b_{it}^{k\alpha_{m}}$ representing, respectively, the minimum number of ambulances of type *k* that must cover the demand area *i* to ensure coverage with reliability *α* and *α*_m_, we solved inequalities (5) and (6) by developing code 1 and code 2 under MATLAB R2020a [2].

Code 1: MATLAB code for computing $b_{\mathrm{it}}^{k}$ values

clear % clean MATLAB memory

alpha = 0.9; % α reliability value declaration

g= [… … …]; % declaration of the row vector containing the values of ρ

A=size(g); % extraction of the size of the vector g

B=A(1,2); % extraction of the number of columns of the vector g

b=ones(B,1); % initialization of b ^k^_it_ values to 1

for i =1:B % a for loop to read the vector g values "vector g contains the values ρ"

k=1;

sigma1 = (g(1,i)^k)/factorial(k);

eq = ((g(1,i)^(b(i,1)))/factorial(b(i,1)))/(1+sigma1); % initialization of the chance constraint ( 5 )

% a while loop to check the satisfaction of the chance constraint ( 5 )

while eq>1-alpha % check if the chance constraint is unsatisfied

b(i,1)=b(i,1)+1; % increment by 1 the value of b ^k^_it_

for k =1:b(i,1) % a for loop to calculate the “sigma” value

sigma = sum((g(1,i)^k)/factorial(k));

eq=((g(1,i)^b(i,1))/factorial(b(i,1)))/(1+sigma); % update of the chance constraint

end

end

end

b % return b "vector containing the values of "b ^k^_it_ "

**Code 2: MATLAB code for computing** $b_{\mathrm{it}}^{k\alpha_{m}}$ **values**

clear % clean MATLAB memory

% declaration of row vectors containing the values of ρ

g1=[… … …]; % decalaration of vector ρ ^k^_it_ (k=1 and t=1)

g2=[… … …]; % decalaration of vector ρ ^k^_it_ (k=1 and t=2)

g3=[… … …]; % decalaration of vector ρ ^k^_it_ (k=2 and t=1)

g4=[… … …]; % decalaration of vector ρ ^k^_it_ (k=2 and t=2)

g=g1; % definition of the vector g which takes, according to the case, the values of g1, g2, g3 or g4

A=size(g); % extraction of the size of the vector g

B=A(1,2); % extraction of the number of columns of the vector g

b=ones(B,90); % initialization of b ^k^_it_ values to 1

% a for loop to read the values of the vector g "the vector g contains the ρ values

for alpha = 1:90

for i =1:B

k=1;

sigma1 = (g(1,i)^k)/factorial(k);

eq = ((g(1,i)^(b(i,alpha)))/factorial(b(i,alpha)))/(1+sigma1); % initialization of the chance constraint ( 6 )

% % a while loop to check the satisfaction of the chance constraint ( 6 )

while eq>1-(alpha/100) % check if the chance constraint is unsatisfied b(i,alpha)=b(i,alpha)+1; % increment by 1 the value of $b_{it}^{k\alpha_{m}}$

for k =1:b(i,alpha) % a for loop to calculate the “sigma” value

sigma = sum((g(1,i)^k)/factorial(k));

eq=((g(1,i)^b(i,alpha))/factorial(b(i,alpha)))/(1+sigma); % update of the chance constraint

end

end

end

end

b1=b;

b1 % return b1 containing the values of $b_{it}^{k\alpha_{m}}$ (k=1 and t=1)

## Ethics Statements

This work did not include data involving human subjects, animal tests, or data acquired from social media platforms.

## CRediT authorship contribution statement

**Youness Frichi:** Conceptualization, Methodology, Software, Data curation, Writing – original draft. **Fouad Jawab:** Methodology, Supervision, Visualization, Validation. **Lina Aboueljinane:** Methodology, Software, Data curation.

## Declaration of Competing Interest

The authors declare that they have no known competing financial interests or personal relationships that could have appeared to influence the work reported in this paper.

## Data Availability

Dataset on ambulance deployment and redeployment (Original data) (Zenodo).

## Footnotes

1 HCP, Annuaire Statistique Régional Fès Meknès, Fès, Maroc, 2019. https://www.hcp.ma/region-fes/attachment/2072183/.

2 http://www.data.gov.ma/data/fr/dataset/la-liste-des-hopitaux
